# 50 Years of INFECTION: happy birthday INFECTION!

**DOI:** 10.1007/s15010-023-02031-w

**Published:** 2023-04-19

**Authors:** Johannes R. Bogner

**Affiliations:** Sektion Klinische Infektiologie, Klinik und Poliklinik IV, Klinikum der Universität München, Pettenkoferstr. 8a, 80336 Munich, Germany

This year we are proud to celebrate the 50th anniversary of INFECTION. I like to congratulate the Journal, the publisher and all persons involved in INFECTION. This is a place to say “thank you” to the founders of the journal, to those who currently read INFECTION and to all those who did so in the past.

INFECTION is grateful to the scientific organizations who are partners, in the forefront of all, the German Infectious Disease Society (DGI, Deutsche Gesellschaft für Infektiologie) along with the partnering societies Deutsche Sepsis-Gesellschaft (DSG), European Society of Clinical Microbiology and Infectious Diseases and Paul-Ehrlich-Gesellschaft für Chemotherapies (PEG).

A lot of work has been invested by our authors, reviewers and editors. I thank you for this and for the good communications and the feedback that helped INFECTION to grow and flourish. Not only reading an article, but also citing manuscripts contributes to the importance and well-being of a scientific journal. The development of one of the most important markers of visibility—the impact factor—shows: over the years INFECTION has improved and gained scientific reputation.

**Fig. 1:**
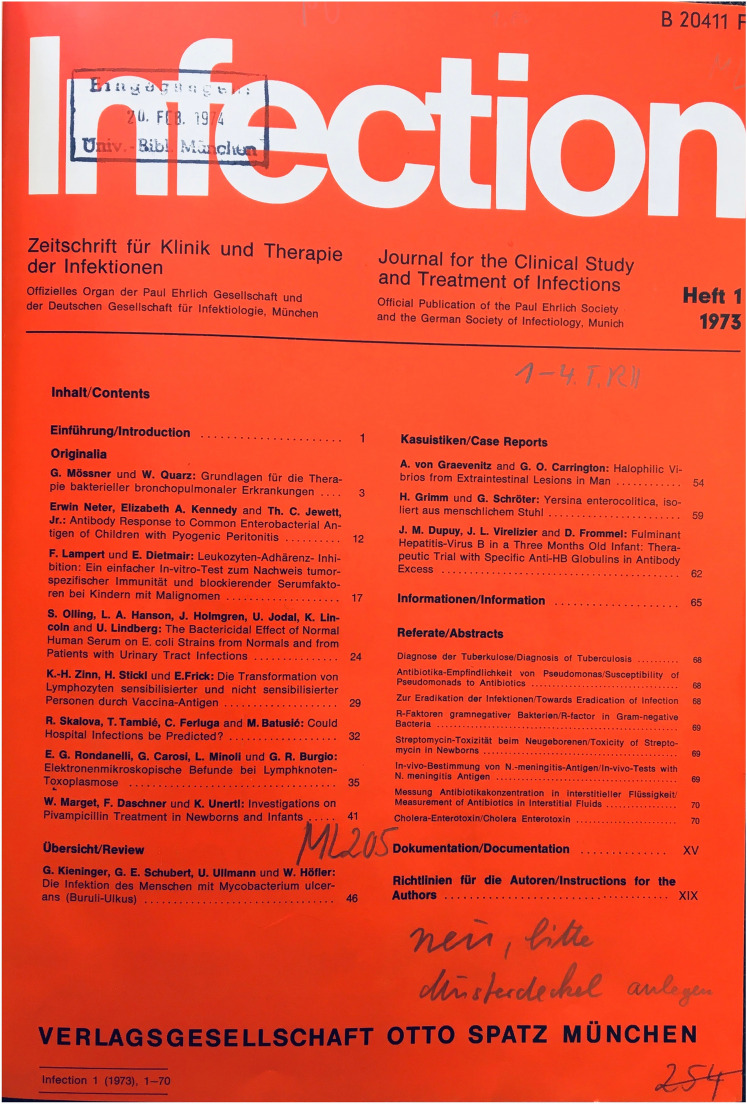
Title page of the first issue of INFECTION in the Year 1973

The journal INFECTION is and always has been a forum for the presentation and discussion of clinically relevant information on infectious diseases for readers and contributors from all over the world. Thus, the journal addresses mainly clinically active infectious disease providers and I always keep the view of the clinician in focus. Our articles have to do with etiology, pathogenesis, diagnosis and treatment of infectious diseases in outpatient and inpatient setting. There is some similarity with the “blue journal”—the nickname of the journal called Clinical Infectious Diseases edited in North America. In contrast to this, the European-based journal INFECTION always has been a journal that came in red colors. Also at the time when it was founded it 1973, its colour was red (see Fig. 1) and the content was one that addresses clinicians and microbiologists as well (Fig. 1 = front page with one of the first tables of content in 1973).

**Fig. 2:**
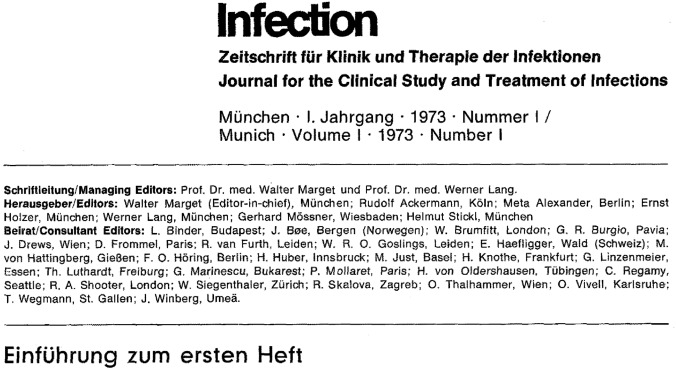
Introductory remarks and list of founders of INFECTION in the first issue

In their introduction to the first issue of INFECTION the managing editors Walter Marget and Werner Lang wrote (Fig. 2) [1]: “The first impression gained might be that this new journal is uncalled for as there are already sufficient periodicals dealing with infectious diseases in man. The concern of the Editors, however, is, basing their considerations on the clinician, to present in this journal current observations, findings and knowledge from a clinical point of view. This journal, introduced by us as the first of its kind in Germany, has evolved from a clinical necessity, which has never before been so forcefully demonstrated as in the recently appearing booklet of 0. Gsell …. The considerable general interest in infectious diseases revealed in numerous articles argues in favour of the appearance of Infection since these papers are either lost among others or address chiefly those who are academically orientated.” [1].

Ten years ago, on 21 January 2013, Walter Marget died at the age of 92 [2]. He was Editor in Chief from 1973 to 1999 and, thereafter, he continued to participate as Senior Editor [3]. In 1999, his successor was Prof. Christian Ruef in Zürich, Switzerland. During his leadership, a transition not only of the publishing house, but also in terms of technical developments toward electronic handling and processing of manuscripts via Editorial Manager was achieved. In several conversations during the years 2011 and 2012, Marget and Ruef accepted my promise to lead the Journal in the spirit of its original mission as a journal of clinical infectious diseases. The intention to bring basic science and clinical care for the patient together resulted in the format of INFECTION and will remain as such in remembrance of the great personality of its founder, Walter Marget. His vision was that “it appeared advisable …. to introduce a journal which serves the whole of Europe in order to draw qualified studies from over a wider area and to promote communication”. Going beyond this vision, the journal is now a journal with international range that serves all continents in a global approach. This is also reflected by an expansion of the board of editors to Asia, Africa and Australia.

Having all this in mind, INFECTION will continue to publish manuscripts dealing with “culture and sensitivity” in the double meaning that is the hallmark of clinical infectious diseases, as originally propagated by Maxwell Finland in the way he defined an infectious disease person’s attributes in specialization and professionalism. Let us continue to bring new aspects of applied microbiology to clinicians and their patients.

This Issue contains several articles that are dedicated to the special occasion of INFECTION’s anniversary. I am also grateful to all authors contributing to the new collection “50 Years of INFECTION” that is the electronic counterpart of an anniversary Issue.

Johannes Bogner.
